# Comparison of vaginal hysterectomy and laparoscopic hysterectomy: a systematic review and meta-analysis

**DOI:** 10.1186/s12905-019-0784-4

**Published:** 2019-06-24

**Authors:** Seung Hyun Lee, So Ra Oh, Yeon Jean Cho, Myoungseok Han, Jung-Woo Park, Su Jin Kim, Jeong Hye Yun, Sun Yi Choe, Joong Sub Choi, Jong Woon Bae

**Affiliations:** 1 Department of Obstetrics and Gynaecology, College of Medicine, Dong-A University, Busan, 26 Daesingongwon-Ro Seo-Gu, Busan, 602-812 Republic of Korea; 2 Department of Obstetrics and Gynaecology, College of Medicine, Hanyang University, Seoul, Republic of Korea

**Keywords:** Laparoscopic hysterectomy, Vaginal hysterectomy, Meta-analysis

## Abstract

**Background:**

There are various surgical approaches of hysterectomy for benign indications. This study aimed to compare vaginal hysterectomy (VH) and laparoscopic hysterectomy (LH) with respect to their complications and operative outcomes.

**Methods:**

We selected randomised controlled trials that compared VH with LH for benign gynaecological indications. We included studies published after January 2000 in the following databases: Medline, EMBASE, and CENTRAL (The Cochrane Library). The primary outcome was comparison of the complication rate. The secondary outcomes were comparisons of operating time, blood loss, intraoperative conversion, postoperative pain, length of hospital stay and duration of recuperation. We used Review Manager 5.3 software to perform the meta-analysis.

**Results:**

Eighteen studies of 1618 patients met the inclusion criteria. The meta-analysis showed no differences in overall complications, intraoperative conversion, postoperative pain on the day of surgery and at 48 h, length of hospital stay and recuperation time between VH and LH. VH was associated with a shorter operating time and lower postoperative pain at 24 h than LH.

**Conclusions:**

When both surgical approaches are feasible, VH should remain the surgery of choice for benign hysterectomy.

**Electronic supplementary material:**

The online version of this article (10.1186/s12905-019-0784-4) contains supplementary material, which is available to authorized users.

## Backgrounds

A substantial number of women undergo hysterectomy annually, and 70 % of hysterectomies are performed for benign indications, including leiomyoma, adenomyosis, severe dysmenorrhea and uterine prolapse [1]. The surgical approach of hysterectomy is the most important factor responsible for postoperative morbidity. Until the present, the approaches for hysterectomies are vaginal, abdominal, laparoscopic and robotic assisted laparoscopic hysterectomy. If feasible, vaginal hysterectomy is associated with a shorter duration of hospital stay, speedier recuperation, fewer unspecified infections or febrile episodes than abdominal hysterectomy [2]. Since Reich first performed laparoscopic hysterectomy (LH) in 1989, various laparoscopic techniques and instruments have been developed, resulting in the vigorous implementation of LH, including laparoscopic-assisted vaginal hysterectomy (LAVH) and total laparoscopic hysterectomy (TLH) at present [3]. In contrast, VH is commonly utilized to treat uterine prolapse, but despite proven safety and effectiveness, it remains underutilized for the surgical treatment of non-prolapse conditions [4]. Gynaecologists perform LAVH or TLH according to their preference, and it is conservative to say that gynaecologists performing LH almost never perform VH [4]. There are several reasons for the widespread implementation of LH. First, LH can facilitate a better anatomical view, which has advantages over VH in cases of severe endometriosis or when there is a history of pelvic inflammatory disease. Second, in cases of large uterine size and for uteruses with little or no descent, LH simplifies the separation of the uterus from its attachment to the pelvic wall [5]. There are multiple approaches to hysterectomy, and each method has its procedure-specific advantages and disadvantages. Since VH and LH are minimally invasive techniques for benign indications that are widely performed around the world, we present a meta-analysis of randomised controlled trials (RCTs) comparing LH with VH for benign gynaecological conditions to identify which surgical approach is superior with respect to various surgical outcomes, especially the rates of complications.

## Methods

### Criteria for considering studies for this review

We selected RCTs that compared VH with LH (LAVH or TLH or unspecified LH) published from January 2000. No language restriction was used. We included women who underwent VH and LH for benign gynaecological indications and excluded women with gynaecological malignancies.

### Study outcomes

The primary outcome of the present analysis was the incidence of intraoperative and postoperative complications. Operative complications were classified by the Dindo classification of surgical complications [6]. Secondary outcomes were operating time, blood loss, rate of conversion to laparotomy, postoperative pain, length of hospital stay and length of recuperation.

### Search methods for studies: electronic searches

This meta-analysis was prepared in accordance with the recommendations of the Preferred Reporting Items for Systematic Reviews and Meta-Analyses Statement (PRISMA Statement) [7, 8]. A literature search for articles published from 1 January 2000 to present was conducted within the main international databases. We searched records from the following databases: Medline, EMBASE, and CENTRAL (The Cochrane Library) for combinations of the terms “hysterectomy,” “laparoscop*,” “vagina*,” “laparoscop*” AND assisted AND vagina*,“and” “benign AND condition*” OR indication* OR disease* OR “disorder*”. Symbol * was used for truncation.

### Data collection and analysis

The studies were included after fulfilling the following inclusion criteria: RCTs; hysterectomy performed for benign gynaecological conditions, and VH outcomes compared with those of any LH. Studies were excluded from the analysis if any one of the inclusion criteria was not met. Two reviewers (SR Oh and SH Lee) independently reviewed the articles and extracted the data. Disagreements were resolved by the other reviewers (JH Yoon, SE Choi). Two reviewers (SR Oh and SH Lee) worked independently and examined the potential eligibility of all the studies retrieved from the database after fulfilling the inclusion and exclusion criteria. Next, they extracted and assessed the risk of bias in each full text article. The other reviewers (JH Yoon, SE Choi) resolved inconsistencies between the first two reviewers through consensus of the whole research team.

### Data extraction and management

First reviewers extracted data from the included studies. The data was confirmed twice by the second reviewers to minimize potential errors. Conflicts were resolved by consensus and discussion. The data extracted from each study included the author, publication year, type of study, number of patients, routes of hysterectomy (VH, LAVH, TLH and unspecified LH), and outcomes (complications, operating time, blood loss, intraoperative conversion, postoperative pain, length of hospital stay and length of recuperation). We first tried to extract numerical data from tables, text or figures. If these data were not reported numerically, we extracted data from graphs using digital ruler software. When summary data included only the median and range, data were transformed according to the methods described by Hozo et al. [9].

### Risk of bias assessment and data analysis

We used tools for assessing quality and risk of bias from the Cochrane Handbook for Systematic Reviews of Interventions to evaluate the methodological quality of RCTs [10]. The following seven items were evaluated:

1. Random sequence generation
2. Allocation concealment
3. Blinding of participants and personnel
4. Blinding of outcome assessment
5. Incomplete outcome data
6. Selective reporting
7. Other bias

The answers for each item included “low” (low risk of bias), “unclear” (either lack of information or uncertainty over the potential for bias), or “high” (high risk of bias). Pairs of independent reviewers assessed the methodological quality. Discrepancies were resolved by consensus of the whole team. A meta-analysis was conducted using Review Manager version 5.3 software, which was designed for and used in Cochrane reviews. Random-effects models were used to calculate a pooled estimate of effect in the meta-analysis. The dichotomous outcomes of each study are represented as the risk ratio (RR) with an estimated 95% confidence interval (CI). The continuous variables are shown as the weighted mean difference (WMD) with 95% CI, which were calculated from the mean, standard deviation (SD), *p*-value, and sample size of each study. Heterogeneity was assessed using Higgins I^2^ value that evaluates the percentage of total variation across a study due to heterogeneity rather than by chance alone: low heterogeneity (I^2^ < 25%), moderate heterogeneity (I^2^ = 25 to 75%), and high heterogeneity (I^2^ > 75%). We used GRADEpro GTD web-based software to rate the quality of each outcome according to GRADE guidelines [11–13].

## Results

**Fig. 1:**
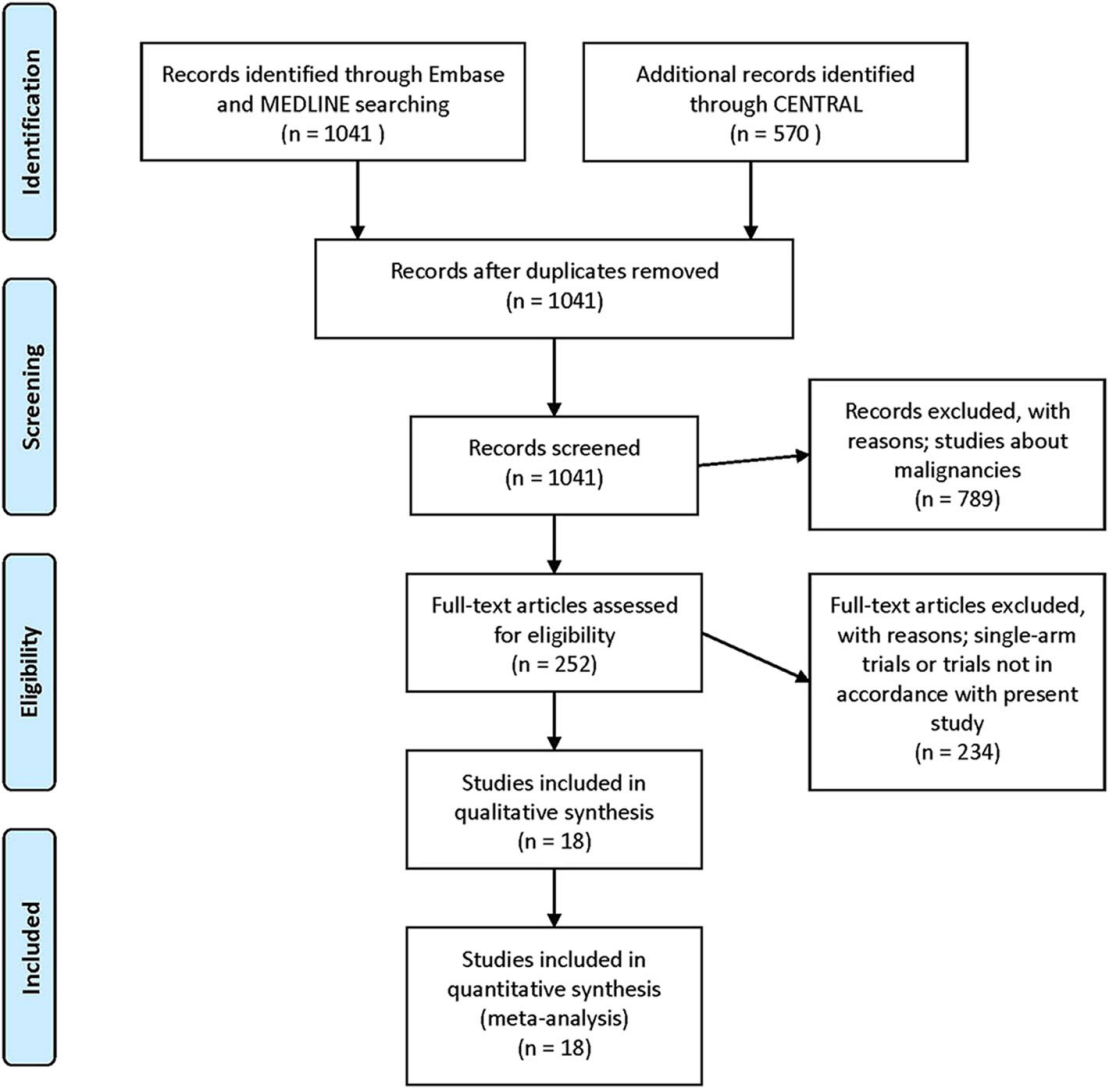
PRISMA flow diagram of the study screening and selection process

**Fig. 2:**
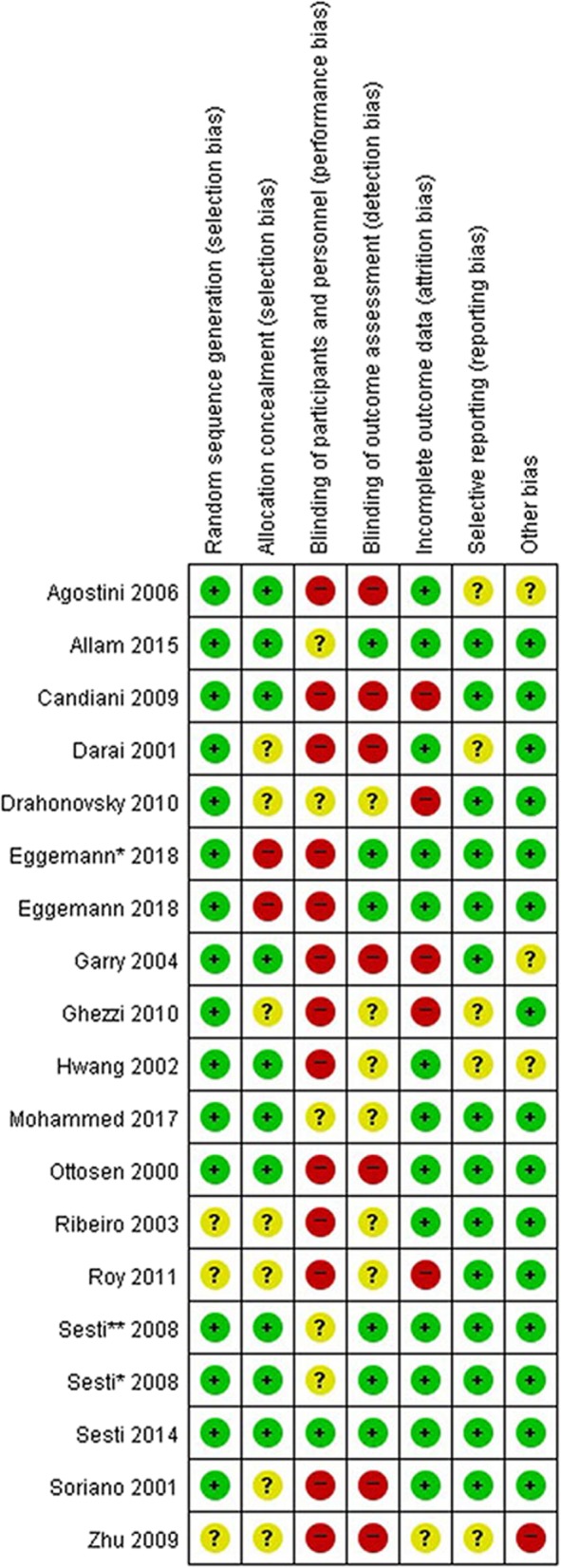
Risk of bias summary. Green circle (low risk), yellow circle (unclear), red circle (high risk)

The primary search retrieved 1611 citations with combinations of the terms “hysterectomy”, “laparoscop*”, “vagina*”, “laparoscop* AND assisted AND vagina*” and “benign AND condition* OR indication* OR disease* OR disorder*”, which were screened for eligible studies. After excluding duplicate citations, 1041 potentially eligible citations were identified and examined in detail. Of these, 1023 articles were excluded because of the inclusion of only one surgical approach (VH or TLH or LAVH), non-RCT design or inclusion of patients with malignancies. Eighteen articles reporting results from RCTs comparing VH (*n* = 677) with LH (*n* = 941) were included in the present meta-analysis (Fig. 1). The meta-analysis was performed using Review Manager, and the studies comparing VH and LH were divided into three subgroups: VH vs. LAVH; VH vs. TLH; and VH vs. unspecified LH. Hence, the number of studies on VH was duplicated in each outcome. The risks of bias in the included studies are summarised in Fig. 2.

### Inclusion/exclusion criteria of studies

Table 1 details the inclusion and exclusion criteria for the 18 studies included in this meta-analysis. Two of the studies specified inclusion of hysterectomy for benign uterine diseases only, and 12 studies included benign uterine diseases and limit of uterine or myoma size. Three studies included benign uterine diseases and possible VH. One included myoma size larger than 8 cm. Four of the included trials excluded women with pelvic organ prolapse (POP) beyond stage I, and eight studies excluded women with pelvic inflammatory disease, endometriosis and/or previous uterine surgeries.

**Table 1:** Characteristics of included studies

First author, year	Type of study	Method	Number of patients	Inclusion criteria	Exclusion criteria	Outcomes	Risk of bias according to Cochrane risk of bias tools
Agostini, 2006	RCT	LAVH vs. VH	48	Benign uterine diseases, uterine size below pubis, favorable to BSO^†^	Adnexal mass	Operative data, complications	high
Allam, 2015	RCT	TAH vs.TLH vs. VH	60	Benign uterine diseases	Malignancy	Operative data, complications	unclear
Candiani, 2009	RCT	TLH vs. VH	47	Benign uterine diseases	Malignancy, estimated uterine volume > 300 mL, POP^‡^, ovarian pathology, PID^§^, endometriosis	Operative data, complications	high
Darai, 2001	RCT	LAVH vs. VH	80	Estimated uterine size > 280 g, contraindications to VH	Malignancy	Operative data, complications	high
Drahonovsky, 2010	RCT	LAVH vs. TLH vs. VH	125	Benign uterine diseases	Impossible VH, prior abdominal surgery, endometriosis, medical disorders	Operative data, complications	high
Eggemann, 2018	RCT	LAVH vs. VH	192	Benign uterine diseases, possible VH	Malignancy, POP^‡^, medical disorders	Operative data, complications	high
Garry, 2014	RCT	Unspecified LH vs. VH	504	Benign uterine diseases	Malignancy, POP^‡^, uterine size > 12 week gestation	Operative data, complications	high
Ghezzi, 2010	RCT	TLH vs. VH	82	Benign uterine diseases	Malignancy, POP^‡^, uterine size > 14 week gestation, large adnexal mass	Operative data, complications	high
Hwang, 2002	RCT	LAVH, vs. TAH vs. VH	60	Myoma > 8 cm	other benign gynecological conditions except myoma	Operative data, complications	high
Mohammed, 2017	RCT	LAVH vs. VH	50	Benign uterine diseases, age (40–70 years), estimated uterine weight < 280 g	BMI > 30, endometriosis, previous myomectomy, medical disorder	Operative data, complications	unclear
Ottosen, 2000	RCT	LAVH vs. TAH vs. VH	80	Benign uterine disease, myoma < 15 cm	Malignancy, uterine size > 16 week gestation, ovarian pathology, dense pelvic adhesion, possible VH	Operative data, complications	high
Ribeiro, 2003	RCT	TAH vs. TLH vs. VH	40	Benign uterine diseases	Estimated uterine volume > 400 cm^3^, medical disorders	Operative data, complications, inflammatory response	high
Roy, 2011	RCT	LAVH vs. TLH vs. VH	90	Benign uterine diseases, estimated uterine weight < 400 g	Malignancy, PID^§^, POP^‡^	Operative data, complications	high
Soriano, 2001	RCT	LAVH vs. VH	77	Estimated uterine size > 280 g, contraindications to VH	Malignancy	Operative data, complications	high
Sesti, 2014	RCT	LAVH vs. TLH vs. VH	108	Symptomatic myoma, age < 55 years, uterine size > 12 week gestation	Malignancy, nulliparity, uterine size > 16 week gestation, previous uterine surgery,	Operative data, complications	low
Sesti, 2008	RCT	LAVH vs. TLH vs. VH	100	Symptomatic myoma, age < 55 years, uterine size > 12 week gestation	Malignancy, nulliparity, uterine size > 16 week gestation, previous uterine surgery,	Operative data, complication	unclear
Sesti, 2008	RCT	LAVH vs. VH	80	Symptomatic myoma, age < 55 years, uterine size > 12 week gestation	Malignancy, nulliparity, uterine size > 16 week gestation, previous uterine surgery,	Operative data, complication	unclear
Zhu, 2009	RCT	LAVH vs. VH	69	Benign uterine diseases	Malignancy	Operative data, complication	high

† Bilateral salpingo-oophorectomy

‡ Pelvic organ prolapse

§ Pelvic inflammatory disease

### Primary outcome

**Fig. 3:**
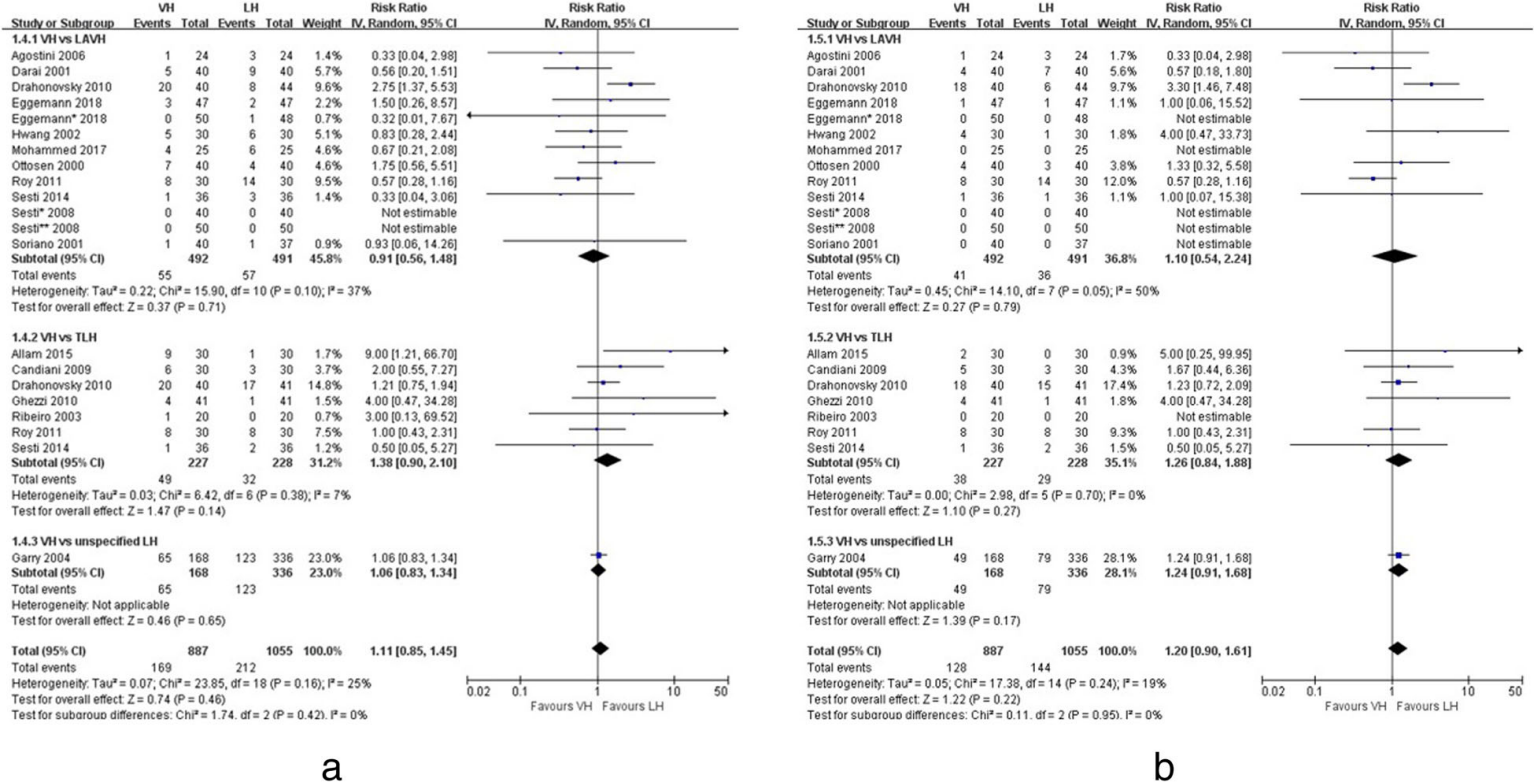
Forest plots of overall (**a**) and grade I (**b**) complications

**Fig. 4:**
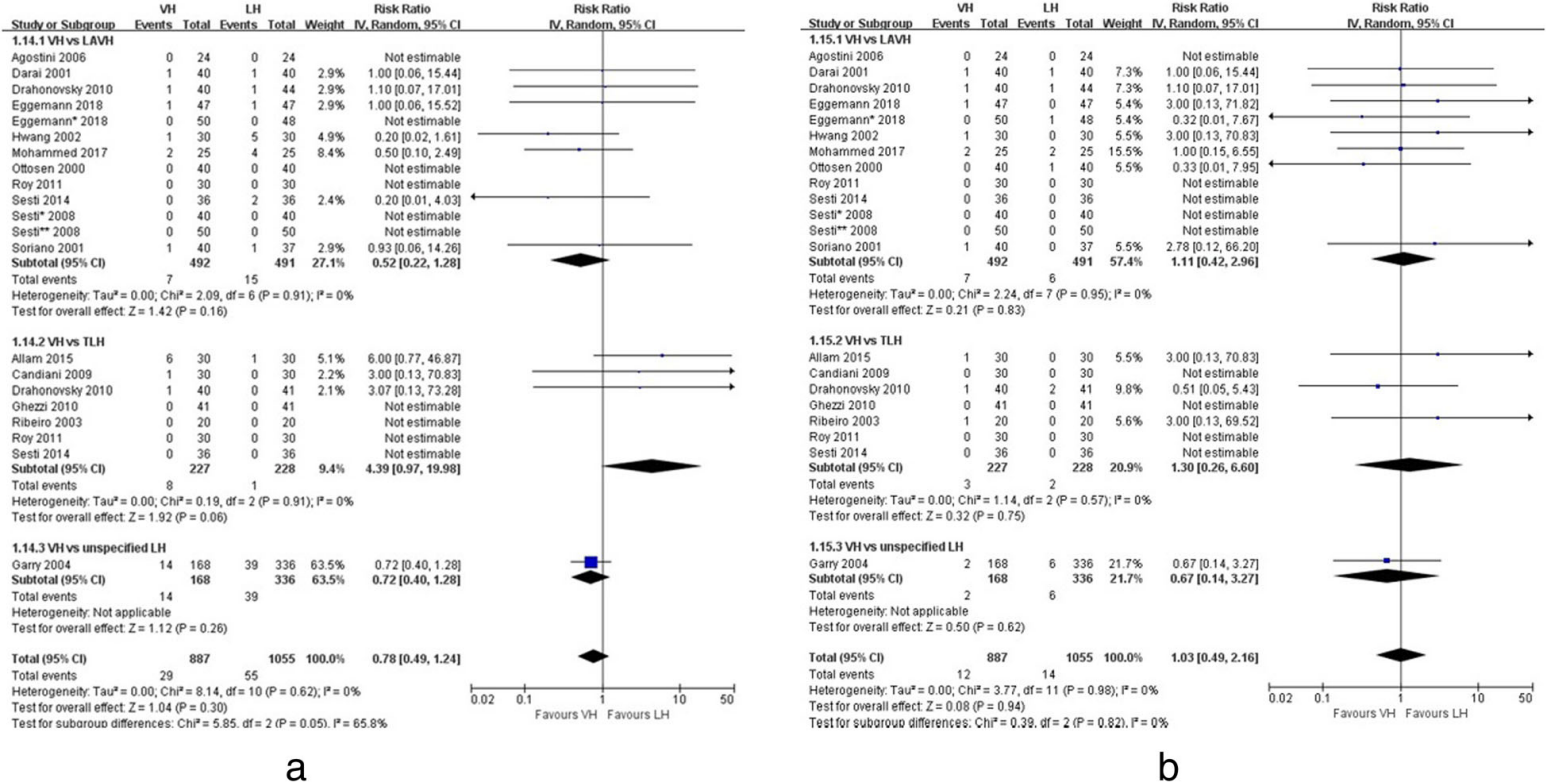
Forest plots of grade II (**a**) and grade III (**b**) complications

Seventeen trials reported incidences of perioperative complications [5, 14–29], which were classified by Dindo classification (grade I to V) [6]. No difference in the rate of overall complications was found between VH and LH (RR 1.11, 95% CI; 0.85 to 1.45, *p* = 0.46). There was also low heterogeneity (I^2^ = 25%) (Fig. 3). Table 2 summarises all the complications in the included studies. Grade I complications were fever, vault hoematoma, urinary tract infection, vaginal bleeding, urinary retention and unspecified infections. No significant differences in the incidence of grade I complications were demonstrated between VH and LH (RR 1.20, 95% CI; 0.90 to 1.61, *p* = 0.22), and there was low heterogeneity (I^2^ = 19%) (Fig. 3). Most of the grade II complications was transfusion (*n* = 82). One patient in the VH group was treated with heparin because of deep vein thrombosis and experienced a spontaneous resolution. No significant difference in the incidence of grade II complications was demonstrated between VH and LH (RR 0.78, 95% CI; 0.49 to 1.24, *p* = 0.30), and there was low heterogeneity (I^2^ = 0%) (Fig. 4). Grade III complications included those requiring surgical, endoscopic, or radiological intervention. There was one ureteral injury, seven bladder injuries and two reoperations in the VH group and eight bladder injuries, one vesicovaginal fistula, one ureterovaginal fistula, one reoperation and two pulmonary embolisms in the LH group. No significant difference in the incidence of grade III complications was demonstrated between VH and LH (RR 1.03, 95% CI; 0.49 to 2.16, *p* = 0.94), and there was low heterogeneity (I^2^ = 0%) (Fig. 4). No significant difference in the incidence of urinary tract injury was demonstrated between VH and LH (RR 1.19, 95% CI; 0.52 to 2.71, *p* = 0.68), and there was low heterogeneity (I^2^ = 0%). None of the trials included in the present analysis reported any grade IV or V complications after either VH or LH.

**Table 2:** Complications by surgical approach

First author, year	VH(n)	Overall complications	Grade I complications	Grade II complications	Grade III complications	LH(n)	Overall complications	Grade I complications	Grade II complications	Grade III complications
Agostini, 2006	24	1	Vault hematoma [1]	0	0	24	3	Fever [1], vault hematoma [2]	0	0
Allam, 2015	30	9	Vault hematoma [2]	Transfusion [6]	Ureteral injury [1]	30	1	0	Transfusion [1]	0
Candiani, 2009	30	6	Fever [5]	DVT^†^ [1]	0	30	3	Fever [3]		
Darai, 2001	40	5	Fever [2], infection [1], vault hematoma [1]	Transfusion [1]	0	40	9	Fever [3], infection [3], vault hematoma [1]	Transfusion [1]	Cystotomy [1]
Drahonovsky, 2010	40	20	Fever [8], vault hematoma [10]	Transfusion [1]	Cystotomy [1]	85	25	Fever [4], UTI^‡^ [3], vault dehiscence [5], vault hematoma [9]	Transfusion [1]	Cystotomy [1] UVF^§^ [1] VVF^§§^ [1]
Eggemann, 2018	97	3	Fever [1]	Transfusion [1]	Cystotomy [1]	95	3	Fever [1]	Transfusion [1]	Cystotomy [1]
Garry, 2004	168	65	Anesthetic problem [1], fever [12], infection [24], vaginal bleeding [2], vault hematoma [10]	Transfusion [14]	Cystotomy [2]	336	123	Anesthetic problem [3], fever [18], infection [36], vaginal bleeding [8], vault hematoma [14]	Transfusion (39)	Cystotomy [3] Pulmonary embolism [2]
Ghezzi, 2010	41	4	Fever [2], urinary retention [2]	0	0	41	1	Urinary retention [1]	0	0
Hwang, 2002	30	5	Fever [4]	Transfusion [1]	0	30	6	UTI^‡^ [1]	Transfusion [5]	0
Mohammed, 2017	25	4	0	Transfusion [2]	Cystotomy [2]	25	6	0	Transfusion [4]	Cystotomy [2]
Ottosen, 2000	40	7	Fever [1], UTI^‡^ [1], vault infection [1], vault hematoma [1]	0	Reoperation [2], cystotomy [1]	40	4	Fever [1], urinary retention [1], vault infection [1]	0	Reoperation [1]
Ribeiro, 2003	20	1	0	0	Cystotomy [1]	20	0	0	0	0
Roy, 2011	30	8	Fever [4], vaginal bleeding [4]	0	0	60	22	Fever [10], UTI^‡^ [4], vaginal bleeding [6], wound infection [2]	0	0
Sesti, 2014	36	1	Fever [1]	0	0	72	5	Fever [2], urinary retention [1]	Transfusion [2]	0
Soriano, 2001	35	1	0	Transfusion [1]	0	37	1	0	Transfusion [1]	0

† Deep vein thrombosis

‡ Urinary tract infection

§ Ureterovaginal fistula

§§ Vesicovaginal fistula

### Secondary outcomes

**Fig. 5:**
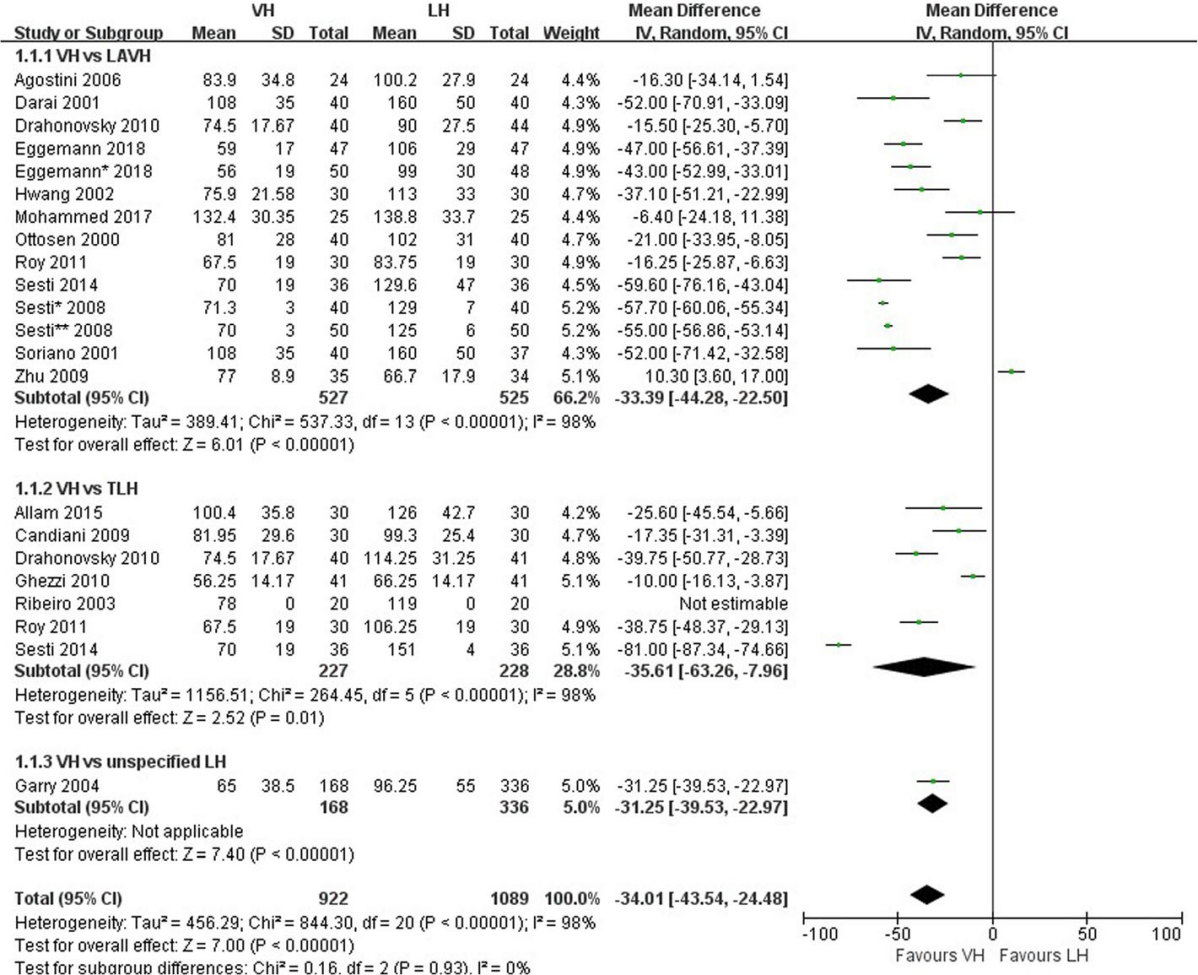
Forest plot of operating time

Secondary outcomes were operating time, blood loss, intraoperative conversion, postoperative pain, length of hospital stay and length of recuperation. Eighteen studies reported on operating time [5, 14–18, 20–31]. VH was associated with a shorter operating time than LH (WMD − 34.01 min, 95% CI; − 43.54 to − 24.48 min, *p* < .0001) (Fig. 5), and there was high heterogeneity between the trials (I^2^ = 98%). However, all studies except one favored VH [31]; thus, the risk of inconsistency for this outcome was not severe. There was no difference in blood loss between VH and LH (WMD − 35.91 mL, 95% CI; − 102.26 to 30.43 mL, *p* = 0.29) in 12 studies [5, 14, 17, 21–29]. There was high heterogeneity (I^2^ = 97%) between trials. Twelve studies assessed intraoperative conversion [14, 17–19, 21, 23–29]. No difference was found between VH and LH (RR 1.16, 95% CI; 0.60 to 2.26, *p* = 0.66), and there was low heterogeneity (I^2^ = 0%). Postoperative pain scores were evaluated using the visual analog scale (VAS) on the day of surgery in four studies [5, 19, 27, 29], at 24 h after surgery in three studies [5, 17, 29] and at 48 h after surgery in three studies [5, 19, 29]. VH was associated with significantly lower VAS pain scores than LH at 24 h after surgery (WMD -0.53, 95% CI; − 0.70 to − 0.35, *p* < .0001, I^2^ = 0%), with low heterogeneity (Fig. 4). There was no difference between the two groups on the day of surgery (WMD 0.80, 95% CI; − 0.08 to 1.68, *p* = 0.07) and at 48 h after surgery (WMD -0.20, 95% CI; − 0.61 to 0.22, *p* = 0.35). Eleven studies reported on the length of hospital stay [14, 17, 19, 21–28]. There was no difference in the length of hospital stay between VH and LH (WMD − 6.57 h, 95% CI; − 18.65 to 5.50 h, *p* = 0.29), and there was high heterogeneity (I^2^ = 99%). Three studies assessed the duration of recuperation [14, 17, 25]. A difference in the recuperation time between VH and LH was not found (WMD 0.65 days, 95% CI; − 6.01 to 7.30 days, *p* = 0.85), and there was high heterogeneity (I^2^ = 92%).

### Assessment of the quality of evidence

We used the GRADEpro GTD web-based software to rate the quality of each outcome according to GRADE guidelines, and the results are summarized in Table 3.

**Table 3:** Rating of quality of evidence with GRADE system

Certainty assessment							Number of patients		Effect		Certainty	Importance
Number of studies	Study design	Risk of bias	Inconsistency	Indirectness	Imprecision	Other considerations	LH	VH	Relative (95% CI)	Absolute (95% CI)		
Overall complications												
17	randomised trials	serious†	not serious	not serious	not serious	none	169/887 (19.1%)	212/1055 (20.1%)	RR 1.11 (0.85 to 1.45)	22 more per 1000 (from 30 fewer to 90 more)	⨁⨁⨁◯ MODERATE	none
Grade I complications												
17	randomised trials	serious†	not serious	not serious	not serious	none	128/887 (14.4%)	144/1055 (13.6%)	RR 1.20 (0.90 to 1.61)	27 more per 1000 (from 14 fewer to 83 more)	⨁⨁⨁◯ MODERATE	none
Grade II complications												
17	randomised trials	serious†	not serious	not serious	not serious	none	29/887 (3.3%)	55/1055 (5.2%)	RR 0.78 (0.49 to 1.24)	11 fewer per 1000 (from 13 more to 27 fewer)	⨁⨁⨁◯ MODERATE	none
Grade III complications												
17	randomised trials	serious†	not serious	not serious	not serious	none	12/887 (1.4%)	14/1055 (1.3%)	RR 1.03 (0.49 to 2.16)	0 fewer per 1000 (from 7 fewer to 15 more)	⨁⨁⨁◯ MODERATE	none
Urinary tract injuries												
17	randomised trials	serious†	not serious	not serious	not serious	none	9/781 (1.2%)	10/1020 (1.0%)	RR 1.20 (0.50 to 2.85)	2 more per 1000 (from 5 fewer to 18 more)	⨁⨁⨁◯ MODERATE	none
Operating time												
18	randomised trials	serious†	not serious	not serious	not serious	none	922	1089	–	MD 34.01 h lower (45.54 lower to 24.48 lower)	⨁⨁⨁◯ MODERATE	none
Length of hospital stay												
14	randomised trials	serious‡	serious§^c^	not serious	not serious	none	636	809	–	MD 6.57 h lower (18.65 lower to 5.5 higher)	⨁⨁◯◯ LOW	none
Blood loss												
16	randomised trials	serious†	serious§	not serious	not serious	none	595	598	–	MD 35.91 mL lower (102.26 lower to 30.43 higher)	⨁⨁◯◯ LOW	none
Intraoperative conversion												
7	randomised trials	serious†	not serious	not serious	not serious	none	15/771 (1.9%)	24/939 (2.6%)	RR 0.94 (0.49 to 1.81)	2 fewer per 1000 (from 13 fewer to 21 more)	⨁⨁⨁◯ MODERATE	none
Recuperation												
4	randomised trials	serious‡	serious§	not serious	not serious	none	130	130	–	MD 0.66 days higher (0.77 lower to 0.9 higher)	⨁⨁◯◯ LOW	none
Pain on day of surgery (assessed with: Visual analogue scale)												
5	randomised trials	serious†	not serious	not serious	not serious	none	325	491	–	MD 0.8 higher (0.08 lower to 1.68 higher)	⨁⨁⨁◯ MODERATE	none
Pain at 24 h after surgery (assessed with: Visual analogue scale)												
4	randomised trials	serious†	not serious	not serious	not serious	none	155	157	–	MD 0.53 lower (0.7 lower to 0.35 lower)	⨁⨁⨁◯ MODERATE	none
Pain at 48 h after surgery (assessed with: Visual analogue scale)												
4	randomised trials	serious†	not serious	not serious	not serious	none	295	461	–	MD 0.2 lower (0.61 lower to 0.22 higher)	⨁⨁⨁◯ MODERATE	none

*CI* Confidence interval, *RR* risk ratio, and *MD* mean difference

† High risk of allocation and blinding

‡ High risk of blinding and incomplete outcome data

§ High heterogeneity

## Discussion

The surgical approach of hysterectomy is the most important factor responsible for postoperative morbidity. Many studies have compared the surgical approach and complications according to the type of surgery to determine which method is best for the patient. The conclusion suggests that abdominal hysterectomy is inferior to VH and LH [32]. There were few randomized trials comparing VH and LH for postoperative complications, operative time, hospital stay, and recovery. The results of our meta-analysis showed no difference between the two groups for the overall rate of complications, including grade I, II and III complications of intraoperative blood loss, intraoperative conversion, length of hospital stay and length of recuperation after surgery. VH was associated with a shorter operative time and less pain at 24 h after surgery than LH. An important matter of concern about LH is a higher incidence of urinary tract injuries [33]. Our meta-analysis showed no significant difference in urinary tract injuries between VH and LH (10 of 887 vs. 10 of 1055; *p* = 0.68). A recent study of 839 women undergoing hysterectomy for benign indications reported that the incidence of urinary tract injuries was 4.3%, including an incidence of 2.9% for bladder injury and 1.8% for ureteral injury [34]. One review article reported that the incidence of ureteral injury is estimated to be 0.03 to 2% for AH, 0.02 to 0.5% for VH and 0.2 to 6% for LH [35]. In this meta-analysis, we found that the incidence of urinary tract injuries was 1.02%. Hence, the incidence of ureteral injury was unlikely to be underreported in the included studies. Interestingly, we found two fistula formations following TLH but no fistula formations following VH. During TLH, many surgeons use electrical laparoscopic instruments to cauterize the uterine artery and dissect the vesicouterine fold; the incidence of fistula formation might thus increase as a consequence of thermal injury [36]. A Cochrane review in 2015 concluded that VH appears to be superior to LH for benign indications, as VH is associated with a faster return to normal activities than LH according to a meta-analysis including two studies of 140 patients [14, 17], and there were no advantages of LH over VH, as the operation time was longer for LH and the incidence of urinary tract injuries was greater for TLH than for VH [32]. Comparing our meta-analysis including four additional RCTs with 440 patients (VH vs. LH) to Cochrane review in 2015, the operation time of VH was significantly faster than that of LH similarly but we found no difference between the two groups in the time to return to normal activities, incidence of urinary tract injury and length of hospital stay. Furthermore, VH was associated with reduced pain scores at 24 h after surgery. The more postoperative pain in LAVH in our study might be caused by the pneumoperitoneum, the pain caused by traction of uterus and the abdominal incisions for the ports [24]. One study concluded that LH was the least cost-effective due to the expensive laparoscopic devices and long operation time [37]. The operation time of LH has shortened over the last couple decades. However, the cost of disposable laparoscopic devices is inevitably more expensive than that of the conventional surgical instruments used in VH.

Gynaecologists around the world should focus on the effect of the rapid development of LH on the treatment of benign indications, especially VH training and skills among residents. When deciding the route of hysterectomy, the preference and proficiency of the surgeon may be the most decisive factors. As a result, if LH is performed more often than VH, gynaecologists in the future will be unfamiliar with VH, leading to a more profound decrease in the implementation of VH. Despite evidence supporting benefits of VH, current statistics indicate VH is underutilised in treating benign gynaecologic conditions [4]. The decreased utilisation of VH is undesirable because VH is the least invasive approach, shorter operating time and less cost than other types of hysterectomy from an evidence-based viewpoint. Main causes associated with decreased utilisation of VH include changes of resident training in surgical techniques due to the tremendous developments of laparoscopic skills and devices, changes of surgical skills in practice, attention to alternative hysterectomy techniques, and enormous propaganda effects of laparoscopic device companies. To increase the rate of VH as the primary approach in possible cases, teaching hospitals around the world should try to increase utilisation of VH on purpose for increasing familiarity with VH during resident training.

According to our review, if both procedures are technically feasible, VH exhibits advantages in the operating time, which can be one of the most important factors for reducing hospital cost. All of hysterectomy cannot be performed by VH, but all of hysterectomy should not be performed laparoscopically.

The limitation of our study is that all included studies had a high risk of bias in blinding despite the RCT design. Hence, no outcome had high-quality evidence according to the GRADE methodology. However, given that our primary outcome was the comparison of complication risk between the two groups, outcomes such as overall complications, grade 3 complications and risk of urinary tract injuries had moderate-quality evidence. Additional large-scale, multicenter, long-term randomized trials including objective outcome assessment will be required to definitively establish the value of LH vs VH.

## Conclusion

The results of this study suggest that VH should be the treatment of benign gynecologic disease when both operative methods are available. Large randomized controlled trials should be performed to identify differences in VH and LH outcomes for operation time, postoperative pain, perioperative complications and cost.

## Additional file

Additional file 1: The raw data of the enrolled studies. (XLSX 12 kb)

## Data Availability

All data generated or analysed during this study are included in this published article and its Additional file 1.

## Abbreviations

AH: Abdominal hysterectomy
CI: Confidence interval
LAVH: Laparoscopic-assisted vaginal hysterectomy
LH: Laparoscopic hysterectomy
RCTs: Randomised controlled trials
RRs: Risk ratios
TLH: Total laparoscopic hysterectomy
VH: Vaginal hysterectomy
WMDs: Weighted mean differences
